# Complex Event Processing for Self-Optimizing Cellular Networks

**DOI:** 10.3390/s20071937

**Published:** 2020-03-30

**Authors:** Isabel de-la-Bandera, Matías Toril, Salvador Luna-Ramírez, Víctor Buenestado, José María Ruiz-Avilés

**Affiliations:** 1 Campus Teatinos, University of Málaga, 29071 Málaga, Spain; mtoril@ic.uma.es (M.T.); sluna@ic.uma.es (S.L.-R.); vbg@ic.uma.es (V.B.); 2 Ericsson, Parque Tecnológico de Andalucía, 29590 Málaga, Spain; jose.maria.ruiz.aviles@ericsson.com

**Keywords:** big data, complex event processing, LTE, mobile network, trace

## Abstract

In a cellular network, signaling and data messages exchanged between network elements are an extremely valuable information for network optimization. The consideration of different types of information allows to improve the optimization results. However, the huge amount of information has made it very difficult for operators to process all the available information. To cope with this issue, in this paper, a methodology for processing cell and user connection traces to optimize a live cellular network is presented. The aim is to generate new performance indicators different from those supplied by manufacturers, taking advantage of the ability of complex event processing tools to correlate events of different nature. For illustrative purposes, an example of how a new performance indicator is created from real traces by complex event processing is given.

## 1. Introduction

Mobile communications have experienced an unprecedented evolution that has led to a rapid increase in the number of mobile users and services. To cope with these changes, new radio access technologies and network architectures have been developed, which has increased the size and complexity of mobile networks. As a result, operators demand automated tools that make network management easier. This demand has stimulated intense research and standardization activity in Self-Organizing Networks (SON) [1]. SON refers to the capability of network elements to self-configure, self-optimize and self-heal. With the arrival of 5G systems, it is expected that the complexity of network management will increase even more [2]. These new networks will bring new challenges that should be addressed [3,4]. In this context, SON will play a key role to deal with the higher heterogeneity of network devices and service requirements [5].

Mobile network management entails the use of a vast amount of information in the form of measurements and control interactions. To properly manage this information, network data is currently separated into performance counters and events. Counters consist of aggregated statistics of network performance, such as, for example, the number of active users, active bearers, or successful/failed HandOver (HO) events. These counters are stored in databases and later used to generate Key Performance Indicators (KPIs) for network benchmarking and optimization. This information is complemented by events, including very detailed information of control messages exchanged by network elements. Such events may be generated by common procedures (e.g., call/session set-up and release) or advanced network functionalities related to SON (e.g., load balancing). Unfortunately, the huge amount of information provided by events has made it very difficult for operators to take advantage of them in network optimization tasks. Current practice is to use events only for identifying the source of problems in the network. Thus, event data is seldom collected in current networks [6]. In the absence of more detailed information, network optimization is currently based on counters. These counters are the result of a long process of specification and implementation by manufacturers. This causes that the set of counters is still limited in new and emerging technologies, such as Long Term Evolution (LTE) and 5G New Radio (NR), which is a major issue for network operators.

To facilitate data gathering, 3GPP has proposed the Minimization of Drive Test (MDT) feature [7]. This functionality allows to collect user measurements more efficiently than by conventional drive tests. Even if this solution covers measurement collection, the resulting data needs to be processed. With the latest advances of information technology, it is now possible to process huge data volumes in real time [8]. This process, known as Big Data Analytics (BDA), aims to uncover hidden patterns, unknown correlations or market trends. In mobile networks, “big data” refers to configuration parameter sets, performance counters, alarms, events, charging data records or trouble tickets [9].

To process network data, several tools have appeared on the market based on Complex Event Processing (CEP). CEP is a data flow management approach operating with data collected continuously, providing a complete solution for event filtering, correlation and processing in (near) real time. CEP methodology was first introduced in Reference [10], where it is shown how events can be used for automatic system management. CEP-based systems allow operators to work with large volumes of heterogeneous information, linking data stored in different locations, generated by different sources at different times. Since then, CEP has been applied in different application domains.

The objective of this work is to apply CEP to mobile communications networks in order to improve the automatic optimization of network performance. Specifically, a methodology is presented for using CEP to generate new performance counters for recently deployed mobile networks. These new counters, referred to as synthetic counters, extend the set of counters currently provided by vendors. Such new counters may combine events and counters generated by different network elements at different instants. Synthetic counters present several advantages for vendors and operators: a) they can be used by vendors to implement temporary counters that can be quickly tested in the field before integrating them in real equipment and b) they can be used by companies offering network optimization services to implement ad-hoc KPIs tailored to the specific needs of an operator or a service provider. Thus, the main contributions of this work can be summarized as: a) an agile methodology for constructing new counters for the radio interface of a mobile network based on CEP, and b) an example of how a new counter is generated from different events in a live LTE network.

The rest of the work is organized as follows. Section 2 presents the state of research and technology in CEP, justifying the novelty of this work. Section 3 outlines the basics of CEP. Section 4 presents the CEP methodology to generate synthetic counters in a mobile network and Section 5 shows an example of use in a live LTE network. Finally, Section 6 summarizes the conclusions of this work.

## 2. Related Work

The basics of CEP are covered in several references. In References [10,11,12], the CEP methodology is introduced, showing how events can be used for automatic management without human intervention. Similarly, Reference [13] describes the event processing technology and capabilities, as well as many of the current commercial applications for event processing. In References [14,15,16], a survey of the event processing tools available on the market is presented. Other references analyze specific event processing tools, such as Cayuga [17] and Eucalyptus [18].

CEP is increasingly used in different application domains. A first domain where CEP is extensively used is financial services. Reference [19] presents an overview of CEP applied to business management and the CEP engine AMiT (Active Middleware Technology). Likewise, References [20,21] show how CEP can be used to monitor and automate business activities. Another application associated with financial services is security. Reference [22] describes the design, implementation and evaluation of a CEP system for detecting credit card fraud, while Reference [23] presents a solution to protect geographically dispersed organizations of a critical infrastructure from coordinated cyber-attacks. Another domain where CEP is applied is service provisioning, such as medical or industrial services. In Reference [24], a CEP system is applied to real time health care. In Reference [25], the use and benefits of CEP applied to the industrial domain is discussed.

While BDA and CEP have long attracted the attention of the computing research community [26], these terms are relatively new in the telecommunications domain. RFID and sensor networks are the first areas where CEP is applied. In Reference [27], an event processing mechanism for an enterprise information systems based on RFID is presented. In Reference [28], a RFID middleware prototype using CEP is described. In Reference [29], a reference architecture for sensor-based networks that process complex event streams in real-time is proposed. In Reference [30], a CEP-based application for object tracking and intruder detection in a wireless sensor network is presented. Similarly, CEP can also be used for data stream processing in Internet of Things (IoT) [31] or match messages from different web services [32].

In spite of its potential, the mobile industry still does not make extensive use of CEP. In Reference [33], an architecture for taking advantage of event-based statistics for real-time performance monitoring and optimization of a Global System for Mobile (GSM) network is described. Later, Reference [34] highlights the large impact that BDA may have on cellular network operators, increasing business opportunities. A recent survey of the technical solutions for the different stages of BDA in large-scale wireless networks is presented in Reference [35], identifying stream data processing as an open research area. BDA has also been recognized as a key enabling technology for 5G networks in the context of SON [6,9]. Reference [6] breaks down the huge amount of data generated by cellular networks that can be used to empower SON algorithms. Likewise, Reference [9] analyzes the requirements of current SON tools to meet 5G requirements, covering machine learning algorithms that can be used to transform raw data into a readily useable knowledge base to create end-to-end intelligence in the network. Moreover, the arrival of 5G networks has brought new scenarios that can be benefited from CEP and BDA, such as IoT [36,37,38], network virtualization [39,40,41] and cloud computing. In these scenarios, security and privacy issues are key challenges to be addressed [42,43].

The interest on BDA has paved the way for applications of event processing in cellular network operation. In Reference [44], a real-time analytics system for cellular networks combining stream and graph processing is presented. The system supports rich and sophisticated analysis tasks based on time-evolving graphs of traffic. With a more limited scope, References [45,46,47] present new performance indicators obtained from events in connection traces for self-tuning and self-healing LTE cellular networks. Nonetheless, to the authors’ knowledge, none of these studies has applied CEP to improve the automatic optimization of cellular networks by taking advantage of the flexibility to generate new performance counters from network data.

## 3. Complex Event Processing

In this section, a general CEP system architecture is introduced. Then, a brief overview of the CEP tool used in this work is given.

### 3.1. Event Processing Architecture

CEP is part of an Event-Driven Architecture (EDA), specifically designed for the production, detection, consumption and reaction to events temporarily ordered and obtained from multiple sources. For this purpose, CEP relies on filtering, matching and aggregating event information in real time.

**Figure 1:**
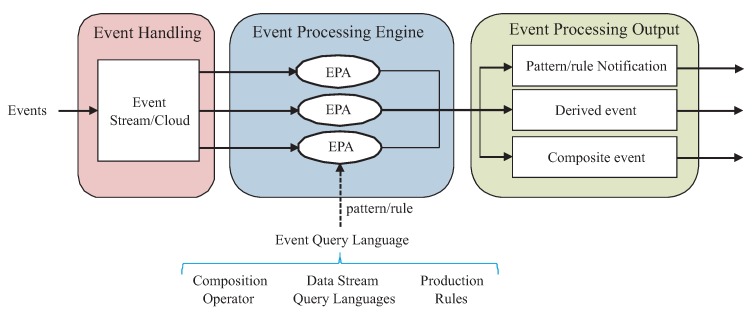
Event processing architecture.

As shown in Figure 1, an event processing system can be separated into three parts: event handling, processing engine and output.

#### 3.1.1. Event Handling

Event processing is usually applied on an already existing information system that provides events as output. An event is anything that happens or is regarded as happening. These events may come from the outside (e.g., from an external database) or the inside (e.g., an internal enterprise information system), and be generated in the real world (e.g., a sensor) or virtually (e.g., by a service).

Events in information systems often include data and messages that record activities. Events can be either simple or complex. A complex event is an event summarizing a set of simple events. For instance, the handover made by a user from a source cell to a target cell is a complex event consisting of several simple events (i.e., user measures the power received by the source and target cells, base station checks if received power from the target cell is better than the power received from the source cell, source cell notifies the outgoing action to the target cell, etc.). Events can be arranged into either an event stream or an event cloud. An event stream is a sequence of events that is linearly ordered by timestamps, denoting the time the event took place [13]. In contrast, an event cloud is a set of event streams obtained from multiple sources. The former is dealt with traditional Event Stream Processing (ESP) tools, while CEP tools also deal with the latter, since CEP can combine data from multiple sources (and hence the term “complex").

In mobile networks, events are collected from many different sources. A source is defined by a combination of event type (e.g., radio measurement, session establishment, handover, …), network element (e.g., base station, site, interface, …) or protocol layer (e.g., physical, Medium Access Control—MAC, Internet Protocol—IP, …). CEP has the ability to combine data from all these multiple sources.

#### 3.1.2. Event Processing Engine

In both event and cloud streams, an Event Processing Engine (EPE) is in charge of processing events. Event Processing Agents (EPAs) are responsible for collecting input events. An EPA can filter duplicate events, rectify errors or match formats [27]. Then, the engine processes events and notifies if certain pre-defined patterns or rules are matched.

A pattern or rule can be defined in an Event Query Language (EQL), which is a high-level programming language for defining complex events. EQLs (a.k.a. Event Processing Languages, EPLs) reduce the effort to develop CEP applications dealing with complicated event patterns in multiple streams. Three main language styles are found for EQL implementation [48]:

1. Composition operator languages (a.k.a. event pattern languages) define complex events by composing single events using different logical operators and nesting expressions (e.g., *ComplexEvent* is equal to *SingleEvent1* and *SingleEvent2*).
2. Data stream query languages (a.k.a. event stream analysis languages) define complex events by converting event data streams into relations, similarly to databases, which are then evaluated by standard Structured Query Language (SQL) queries. The resulting relations are converted back to another data stream.
3. Production rule languages define complex events by specifying the actions to be executed when certain states are reached. These states are defined by means of “WHEN … THEN …" rules (e.g., WHEN *SingleEvent1* AND *SingleEvent2* THEN *ComplexEvent*).

Nowadays, SQL-based data stream query languages are the most successful approach, since they can easily be integrated with databases, sharing the common basis of SQL. Nonetheless, some tools combine the three programming styles to benefit from their strengths.

Retaking the handover example, the EPE can define a set of statements to process not only the events related to the handover, but also other events received by the base station at the same time (e.g., signal strength measurements periodically reported by the terminal for the source and target cell, instantaneous load measurements periodically taken by the serving cell, interference issues reported asynchronously by neighbor cells, …).

#### 3.1.3. Event Processing Output

Two actions can be triggered from the output of the EPE. On the one hand, the engine can only notify that a given pattern or rule has been detected, and maybe update some counter. Alternatively, a new event can be generated. The new event can be a composite or a derived one, depending on the information to be included in the new event. A *composite* event is created when the information is selected from the information in original single events. Instead, a *derived* event includes new information, not existing in the previous single events, and specifically collected when the new event is triggered.

In the handover case, a new event can be generated based on the information included in all considered events in previous stages. This example is described in more detail in Section 5.

### 3.2. Cep Software

Table 1 lists some of the most popular CEP tools on the market. These can be classified into Event Processing Platforms (EPP), Distributed Stream Computing Platforms (DSCP) and CEP libraries (CEPL) [16]. EPPs are platforms that provide high-level programming models built-in functions for event filtering, correlation, and abstraction. DSCPs are platforms that provide explicit support for distribution of computation across multiple nodes in a computing cluster. CEP libraries (a.k.a. as CEP engines) are isolated lightweight software components focused on detecting complex events, which can be integrated into EPPs and DSCPs.

Some commercial products (e.g., IBM Operational Decision Manager, IBM InfoSphere Streams, SAP Event Stream Processor, Tibco BusinessEvents and StreamBase) are runtime software suites with adapters for dashboard, alerting and administration tools. Other event processing platforms (e.g., FeedZai Pulse, SQLStream s-Server) are extended with features, such as query, reporting, interactive analytics or key performance indicators, specifically aiming at operational intelligence applications. Emerging distributed systems (e.g., Apache Samza, Spark and Storm) are general-purpose environments without native CEP functions, but offering high scalability and extensibility. The reader is referred to Reference [16] or Reference [49] for a detailed comparison of their features.

The Esper [50] suite is one of the few open-source software packages for CEP available in the public domain. Developed in JAVA, Esper can be integrated into different platforms or used as a standalone container. It combines traditional and complex event processing (i.e., ESP and CEP) and offers an SQL-based data stream query language, which makes integration with databases easier.

## 4. Complex Event Processing in Mobile Networks

The use of real network data is key to improve network optimization algorithms. In this section, a CEP-based framework is presented to derive new performance counters suitable for self-optimization of mobile radio access networks. These synthetic counters are obtained from trace files reported by base stations (BS), and can be used to enrich the information provided by vendors’ traditional counters.

A general CEP approach for mobile networks consists of three stages: event decoding, synchronization and correlation. For clarity, the trace collection process in existing mobile networks is explained first, and the different event processing stages are detailed later.

### 4.1. Traces

In mobile networks, network management data is distributed across different elements. Depending on its nature, data in the radio interface can be classified into:

1. Configuration Management (CM) information, reflecting the current value of network parameter settings (e.g., maximum transmit power of a BS);
2. Performance Management (PM) information, consisting of counters reflecting the number of times that some event has occurred in a network element (e.g., number of connection establishment attempts) during a certain period, referred to as Reporting Output Period (ROP); and
3. Data Trace Files (DTFs), containing multiple records (events) with radio related measurements of a single User Equipment (UE) or a base station when some event occurs (e.g., received signal level when a connection starts). This information is gathered by MDT function. DTFs can be further classified into User Equipment Traffic Recording (UETR) and Cell Traffic Recording (CTR) [51]. UETR are used to monitor a specific user, while CTR are used to monitor cell performance by monitoring multiple and anonymous connections. Note that both UETR and CTR consist of traces of individual connections. The main difference between UETR and CTR traces is that, in UETR, it is the operator that decides which UE is tracked, whereas, in CTR, all (or a random subset of) UEs in a cell are recorded (i.e., it is not possible to single out a particular user)) [52]. A DTF includes events from multiple sources, such as UEs or cells.

Events can be classified in two groups, depending on the network entities involved:

1. External events, consisting of signaling messages that BSs exchange with other network elements. For instance, in LTE, BS (a.k.a., eNodeB, eNBs), store Radio Resource Control (RRC) messages received from the UE through the LTE-Uu interface, and messages exchanged with other eNBs through either the X2 or the S1 interface. Therefore, external events can be divided into three categories depending on the type of message:
  (a) RRC events (e.g., Rrc_rrc_connection_request or Rrc_rrc_connection_setup),
  (b) S1 events (e.g., S1_initial_context_release or S1_initial_context_setup) or
  (c) X2 events (e.g., X2_handover_request or X2_handover_request_acknowledge).
2. Internal events, with information about the performance of some BS. These events are specific to each BS vendor. Some examples of internal events are:
  (a) Periodical events, including information about user or BS performance (e.g., periodic pilot signal level/quality measurements).
  (b) Non-periodical events, triggered by some sporadic reason (e.g., start/end of a connection, handover).
  Both periodical and non-periodical events can be divided into UE or BS events.

The structure of events stored in DTFs is usually made up of a header and a message container that includes different attributes, referred to as event parameters. The header contains general attributes associated with the event description, such as timestamp, BS, UE, message type or event length, whereas the message container includes specific attributes associated with the message type. The number of attributes in the message container depends on the message type.

**Figure 2:**
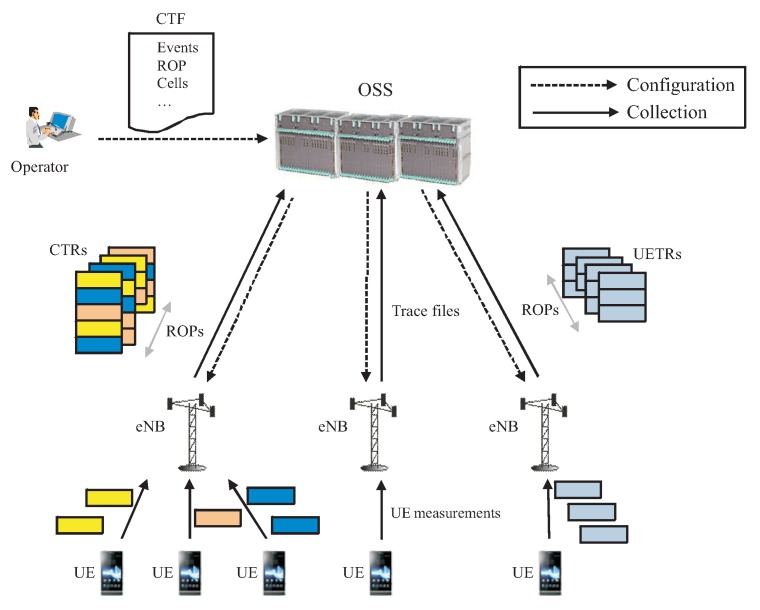
High-level view of trace reporting.

Figure 2 represents a high-level view of the architecture for trace reporting [52]. The trace collection process starts by the operator preparing a Configuration Trace File (CTF). A CTF includes: (a) the event(s) to be monitored, (b) in the case of UETR, the particular UE(s), and, in the case of CTR, the cells and the ratio of calls, for which traces are collected, (c) the ROP (typically 15 min), (d) the maximum number of traces activated simultaneously in the network, and (e) the time period when trace collection is enabled. After enabling trace collection, UEs transfer their event records to their serving BS. When ROP is finished, the BS generates CTR and UETR files, which are stored locally. Later, trace files are periodically sent to the Operations Support System (OSS) or another trace collection entity owned by the operator. CTF parameters must be configured properly to avoid overloading network elements, especially when dealing with high-frequency events (e.g., periodic measurements on a connection basis). Note that DTFs have a limited size. Thus, a CTF activating too many records in a cell might cause that the maximum DTF size is reached before the ROP ends, causing that some connections were not traced properly.

### 4.2. Event Decoding

Trace files are binary files encoded in ASN.1 format [51]. For computational efficiency, the DTF from each BS is broken down in as many files as event types. These temporary files are then converted into a common file format, for example, Comma Separated Values (CSV) file. Such a decoding is performed by a parsing tool that extracts the information in traces. The output of this stage is one file per event type, BS and ROP, which is the input to the synchronizer block.

### 4.3. Event Synchronization

Once traces have been decoded and separated by event type, BS and ROP, data is synchronized (i.e., ordered by time). Such a process consists of merging all input files into a single file per event type, and then sorting events by the *timestamp* attribute. As a result, a file is obtained per event type including all the events of the same type reported by all BSs during the trace activation period, sequentially ordered by time.

### 4.4. Event Correlation

The event correlation process is the core of the analysis. In the proposed framework, the correlation engine is implemented by Esper [50]. The input to Esper is the set of decoded and synchronized traces from previous stages, consisting of a file per event type with events sorted by timestamp. Each of these files represents an event stream. From these files, Esper derive and aggregate information by defining EPL statements similar to SQL.

In Esper, data is processed in a continuous manner. Once EPL statements are registered, these are executed as live data streams are introduced into the tool. The output of a statement can be the input of another statement. This is done by a listener, which propagates query results acting as an internal input.

**Figure 3:**
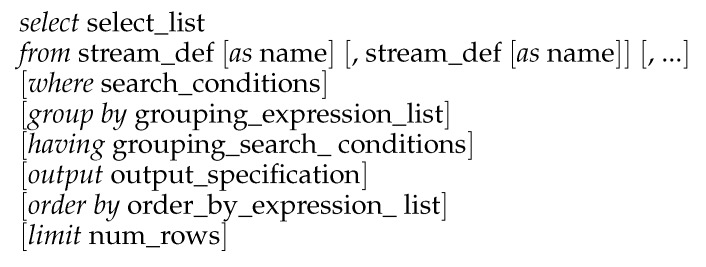
EPLstatement.

Figure 3 shows the structure of a typical EPL statement. The *select* clause indicates the analyzed event attributes, while the *from* clause indicates the analyzed event streams. For ease of use, a short name can be assigned to event streams or attributes with the operator *as*. The *where* clause defines the event attribute (or combination of them) to search for. The *group by* clause is used to arrange identical data into groups. The *having* clause adds filtering conditions for the *group by* clause. The *output* clause is used to control the rate at which events are supplied by the event correlation engine. The *order by* clause sorts data in ascending or descending order. Finally, the *limit* clause is used together with the *order by* and *output* clauses to limit the query results to those within some specific range. The *select* and *from* clauses are mandatory, whereas the rest of clauses are optional (expressed in square brackets). Likewise, the order of clauses must be maintained.

A valuable feature supplied by EPL statements is the definition of moving event windows. A window can be defined by a period duration or by a number of times an event occurs (referred to as logical or physical windows, respectively). A window can also be set as sliding or tumbling. A sliding window extends the specified interval into the past from the present moment, reporting an event as it occurs. In contrast, a tumbling window batches events and reports them only when the window is closed. Consequently, tumbling windows report only at the end of the time period (logical tumbling windows) or after some event occurs a predetermined number of times (physical tumbling windows) [50].

## 5. Use Case

An example of how a synthetic counter is implemented with CEP is presented next. The aim is to show the ability of trace processing to generate sophisticated indicators for mobile network optimization. Data in the example is taken from a live LTE network. For clarity, the design of the synthetic counter is first explained and results are presented later.

### 5.1. Synthetic Counter Design

The implemented counter computes the average pilot signal level (i.e., Reference Signal Received Power, RSRP) received by UEs just before a HO, on a per-adjacency basis. This is achieved by combining information at cell, user and connection level.

**Figure 4:**
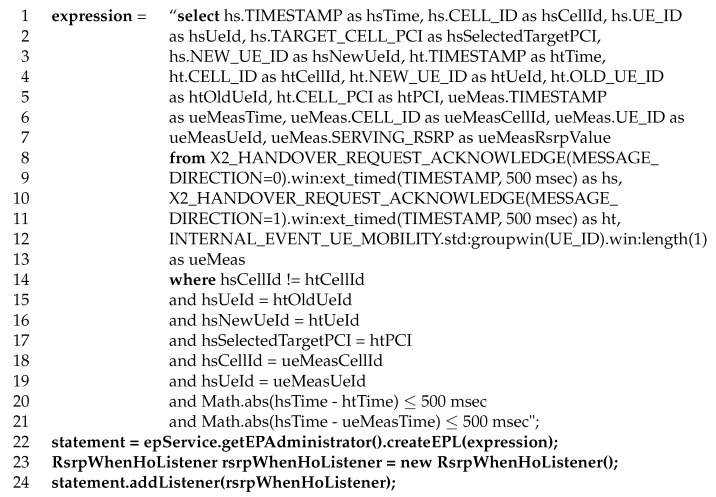
Esper code for the synthetic counter *AverageRSRPwhenHO*.

The implementation of a counter includes three components: (a) the event streams and attributes required as an input, (b) the correlation rules between different event streams, and (c) the actions to be carried out when rules are met. Figure 4 shows the Esper code that implements the proposed synthetic counter following the structure in Figure 3. The defined structure includes the definition of the EPL statement with its streams, attributes and rules (lines 1–15 in Figure 4), the creation of the EPL statement (line 16 in Figure 4), the definition of output actions (line 17 in Figure 4) and the activation/registration of the EPL statement (line 18 in Figure 4).

#### 5.1.1. Event Streams

In the code in Figure 4, the input event streams are defined in the variable “**expression**” in the **from** clause (lines 8–13). Two event streams are invoked: the external X2_HANDOVER_REQUEST _ACKNOWLEDGE and the internal INTERNAL_EVENT_UE_MOBILITY. Both input streams consist of a series of records from the eNB. A new record is saved by an eNB in the external X2_HANDOVER_REQUEST_ACKNOWLEDGE event stream when either an incoming or an outgoing HO is requested. Likewise, a new record is saved by an eNB in the internal INTERNAL_EVENT_UE_MOBILITY event stream when a connection is released. In the example, the **from** clause consists of three components. The former four (lines 8–11) deal with the X2_HANDOVER_REQUEST_ACKNOWLEDGE. The first ones (lines 8–9) corresponds to an outgoing HO request in the source cell (indicated by MESSAGE_DIRECTION=0), while the second ones (lines 10–11) corresponds to an incoming HO request in the target cell (MESSAGE_ DIRECTION=1). These two components are renamed as *hs* and *ht* (for HO events from source and target cells, respectively). In both, a sliding window is configured by means of the method “win:ext_timed(TIMESTAMP, 500 msec)", that is similar to the sliding window, but based on the millisecond time value supplied by the expression. Thus, the *from* clause is activated when a HO is detected from a source cell to a destination cell in the last 500 msec. The third one (line 12) deals the INTERNAL_EVENT_UE_MOBILITY stream, associated with measurements reported by the UE, and hence renamed as *ueMeas*. In this stream, the methods “std:groupwin(UE_ID)" and “win:length(1)" are used to single out events from a particular UE.

#### 5.1.2. Event Attributes

The attributes analyzed in every record in the event streams are listed in **expression** by the **select** clause (lines 1–7). Table 2 gives a detailed description of these attributes.

#### 5.1.3. Conditions

A set of conditions to be met by the selected attributes of events to trigger an action are given by the **where** clause in **expression** (lines 14-21). Table 3 gives a detailed description of these conditions.

#### 5.1.4. Actions

Once the EPL statement is registered, the framework continuously checks for matches. Every match triggers plain Java or .Net/C# objects for customized actions. In the example, actions are defined inside the **RsrpWhenHoListener** class (line 23). In this case, actions consist of updating two intermediate counters with the aggregated RSRP level and the number of measurement samples on a per-adjacency basis. These counters are then used to compute the final synthetic counter with the average RSRP just before HO for each adjacency, hereafter denoted as *AvgRSRPWhenHO*.

### 5.2. Assessment Methodology

The above described Esper code is executed over a trace dataset collected in a live LTE network. The geographical area covered by the dataset consists of 145 eNBs and 3220 adjacencies, covering 835 km${}^{2}$ of a mixture of urban and residential areas. The dataset includes 580 CTR files collected from sites every 15 min during the busy hour of a weekday (i.e., 4 ROPs of 15 min per cell, leading to 145×4=580 files). To avoid overloading eNB processors, a limited share of connections is traced per cell. For this purpose, the CTF is set to monitor 20% of connections. Posteriori, field tests show that tracing 20% of connections is enough to obtain robust statistics. The total number of traced connections is 2332961. For operational reasons, the event processing tool is not executed in real time, but after the CTR files are uploaded in the OSS.

The proposed synthetic counter is compared with the two indicators currently used by operators to monitor coverage issues—the average RSRP and the 5th percentile of the RSRP distribution per cell, showing the average (cell center) and worst-case (cell edge) signal strength conditions, respectively [53]. Both indicators are calculated from counters that register the bins of the histogram of the RSRP distribution per cell, derived by aggregating all users in a cell. These counters are provided by all vendors.

### 5.3. Results

**Figure 5:**
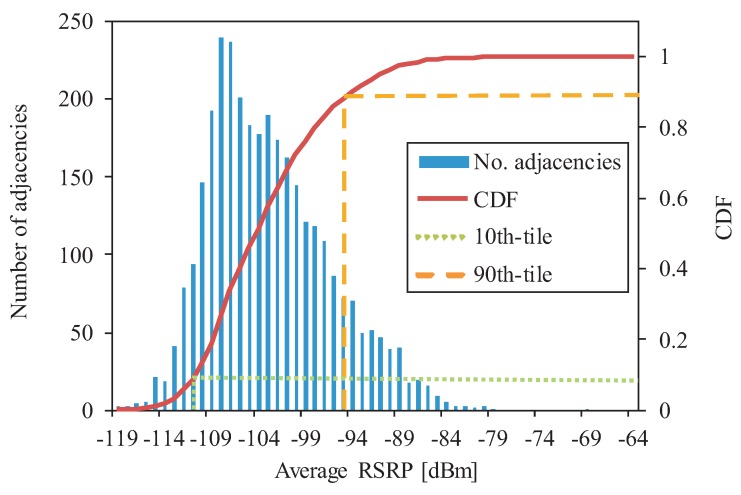
Probability distribution of *AvgRSRPWhenHO* synthetic counter.

As described before, a different value of *AvgRSRPWhenHO* per adjacency is obtained. Figure 5 presents the histogram (bars) and Cumulative Distribution Function (curve) for the *AvgRSRPWhenHO* indicator, computed on an adjacency basis (1 value per adjacency). For clarity, the 10th and 90th percentiles are marked by a dashed and dotted lines (−111 and −95 dBm, respectively). Some relevant findings can be drawn from the figure. A preliminary analysis of network settings (not presented here) showed that the operator set the RSRP threshold to drop a call to −116 dBm for all adjacencies. When this value is compared with the *AvgRSRPWhenHO* percentiles shown in Figure 5, it is concluded that, in 10% of adjacencies in the network, users experience a RSRP value before HO lower than −111 dBm, which is too close to the call dropping threshold, −116 dBm. Thus, it is concluded that, in that 10% of adjacencies, handovers are triggered too late. This is normally due to a coverage problem at cell edge, reflected by the low RSRP before HO in the adjacency. Likewise, the 90th *AvgRSRPWhenHO* percentile shows that 10% of adjacencies in the network have a RSRP value before HO larger than −95 dBm. This threshold can be used to detect adjacencies that might be triggering unnecessary HOs, since UEs experience good RSRP before HO at the source cell. Such situations might be indicative of excessive cell overlapping, which would generate high interference due to the target cell radiating the service area of the source cell with a strong signal level. Moreover, useless handovers unnecessarily increase network signaling load.

**Figure 6:**
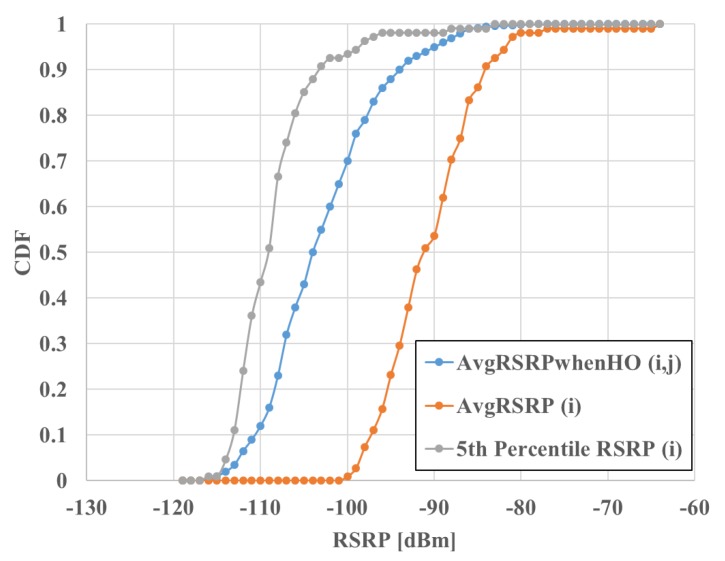
Comparison of *AvgRSRPWhenHO*, average RSRP (Reference Signal Received Power) and 5th percentile of RSRP.

To show need for the synthetic counter, Figure 6 compares the cumulative distribution function of *AvgRSRPWhenHO* per adjacency against the average RSRP and 5th percentile of the RSRP distribution per cell. It is observed that none of the two indicators derived from standard counters can approximate the signal level received by the user around the handover event. Not shown is the fact that the latter two are also affected by the granularity of histogram bins, which is not the case for the synthetic indicator, as it is not computed from histograms. Moreover, note that the latter two can only be computed on a cell basis, unlike the synthetic counter, which has been specifically designed to be computed on an adjacency basis. Thus, the new counter provides directional information that can be used to steer base station antennas.

### 5.4. Implementation Issues

Both the event decoding and synchronization stages were developed in C++, while the event correlation block was developed in JAVA with Esper. The theoretical time complexity of event processing is $O(N_{c})$, where $N_{c}$ is the number of connections collected per ROP. In practice, event decoding, synchronization and correlation took 4 min, 10 and 50 s, respectively, for a total execution time of 5 min, in a laptop with 2.6 GHz frequency clock and 4 GB of RAM, for 1 h of traces.

### 5.5. Limitations

The proposed approach has two limitations. On the one hand, synthetic counters still rely on the signaling events provided by vendors. Note that the proposed counter is derived from the combination of an external (standardized) event and an internal (non-standardized) event. To ensure that different vendors can reuse the design of a synthetic counter, this should be computed only from data fields in external (i.e., vendor-independent) events. This constraint is important when optimization schemes are executed in multi-vendor scenarios. On the other hand, the proposed synthetic counter is computed offline in a centralized entity for simplicity. Thus, it is possible to correlate/aggregate events that can be very distant in space and time. If the value of the counter has to be computed in real time (e.g., for online monitoring of individual connections), a careful analysis of the different sources of delay (e.g., averaging windows, information exchange between network elements, computational load …) has to be performed to ensure that the result is available in due time.

## 6. Conclusions

In this work, a generic methodology has been presented for building new performance counters for monitoring mobile networks by processing events in connection traces in the radio interface. The resulting indicators, referred to as synthetic counters, enrich the set of counters provided by vendors. The power of counters generated by CEP lies on the ability to correlate events at cell, user and connection level, combining information from different network elements and time instants, collected with different space and time granularity. Equally important, event correlation rules are configured at run-time, so that counters can easily be modified. As a proof of concept, a CEP-based framework based on Esper suite has been developed to compute an indicator for the performance of the handover mechanism in a live LTE network.

The capability of adapting the set of performance counters will be extremely valuable in future 5G systems. It is envisaged that one of the key benefits of 5G will be to reduce time to market for new functionalities and services [54]. To make it true, a flexible performance monitoring solution that can be adapted to the different stages in the technology roadmap is crucial. Performance data needed to verify a product in the lab, or tune a golden cluster in a field trial, differs from the information used for large scale monitoring in the operational stage, when traffic demand increases and aggregated counters truly reflect network performance [55]. Likewise, network tuning and troubleshooting tasks, currently offered as consultancy services or automation features, often require very detailed information not provided by default counters [6]. These differences call for an agile network monitoring solution including fast prototyping of new performance counters.

In 5G systems, the isolation of tenants provided by network slicing will be key to cope with the expected service diversity [56]. For this purpose, network performance counters must reflect end user experience to ensure that operator policies have the largest impact on subscribers. The methodology proposed here can be applied to merge information from different radio access technologies and domains (subscriber, radio, transmission, core network…) to allow end-to-end performance monitoring of individual users in real time per slice [57]. Moreover, the ability of quickly redesigning the counter set might help operators to define more specific service level agreements, which is impossible in legacy systems due to the cost of developing new counters with the traditional approach. This capability can be extended to tenants (e.g., vertical industries or virtual operators), who could tailor service measurements to their needs. Ultimately, the availability of analytical tools as the one proposed will help operators to convert operational data into actionable insights.

## Figures and Tables

**Table 1:** Complex event processing tools available on the market.

Tool	Developer	Class	Type
Apama	Software AG	EPP	Commercial
BusinessEvents	Tibco	EPP	Commercial
StreamBase	Tibco	EPP	Commercial
InfoSphere Streams	IBM	EPP	Commercial
SAP Aleri Streaming	Sybase	EPP	Commercial
SQL Server StreamInsight	Microsoft	EPP	Commercial
Oracle CEP	Oracle	EPP	Commercial
SQLStream	SQLStream	EPP	Commercial
Complex Event Processor	WSO2	EPP	Free
Kinesis	Amazon	EPP	Free
DataTorrent RTS	Apache	EPP	Free
Stream Explorer	Oracle	EPP	Free
Borealis (Aurora, Medusa)	Brandeis University, Brown University and MIT	DSCP	Free
Storm	Apache	DSCP	Free
Spark Streaming	Apache	DSCP	Free
Samza	Apache	DSCP	Free
Apex	Apache	DSCP	Free
Flink	Apache	DSCP	Free
Active Middleware Technology	IBM	CEPL	Commercial
Esper/NEsper	EsperTech	CEPL	Free
Cayuga	Cornell University	CEPL	Free
Siddhi	Cornell University	CEPL	Free
ruleCore CEP Server	Rulecore	CEPL	Free

**Table 2:** Event attributes.

Attribute	Renamed as	Information
hs.TIMESTAMP	hsTime	Time when record is saved in event stream of source cell
hs.CELL_ID	hsCellId	Logical cell identifier of source cell in event stream of source cell
hs.UE_ID	hsUeId	UE identifier in source cell in event stream of source cell
hs.TARGET_CELL_PCI	hsSelectedTargetPCI	Physical cell identifier of target cell in event stream of source cell
hs.NEW_UE_ID	hsNewUeId	UE identifier in target cell in event stream of source cell
ht.TIMESTAMP	htTime	Time when record is saved in event stream of target cell
ht.CELL_ID	htCellId	Logical cell identifier of target cell in event stream of target cell
ht.NEW_UE_ID	htUeId	UE identifier in target cell in event stream of target cell
ht.OLD_UE_ID	htOldUeId	UE identifier in source cell in event stream of target cell
ht.CELL_PCI	htPCI	Physical cell Identifier of target cell in event stream of target cell
ueMeas.TIMESTAMP	ueMeasTime	Time when record is saved in event stream of UE measurements
ueMeas.CELL_ID	ueMeasCellId	Logical cell identifier of cell serving in event stream of UE measurements
ueMeas.UE_ID	ueMeasUeId	Identifier of UE reporting measurement in event stream of UE measurements
ueMeas.SERVING_RSRP	ueMeasRsrpValue	Reference signal received power level from serving cell reported by UE in event stream of UE measurements

**Table 3:** Conditions.

Condition	Description
hsCellId != htCellId	Source and target cells are not the same
hsUeId = htOldUeId and hsNewUeId = htUeId	UE triggering outgoing HO in the source cell is the same as UE triggering incoming HO to target cell
hsSelectedTargetPCI = htPCI	Physical cell identifier of target cell in outgoing HO is the same as physical cell identifier in the incoming HO
hsCellId = ueMeasCellId	Cell where outgoing HO is triggered is the same as cell where UE measurements are reported at the end of the connection
hsUeId = ueMeasUeId	UE triggering outgoing HO from source cell is the same as UE reporting measurements at the end of its connection
Math.abs(hsTime − htTime) ≤ 500 msec and	Absolute time gap between *hs* and *ht* and between *hs* and *ueMeas* events
Math.abs(hsTime − ueMeasTime) ≤ 500 msec	must be less than 500 ms
